# Metabolically active bacteria detected with click chemistry in low organic matter rainwater

**DOI:** 10.1371/journal.pone.0285816

**Published:** 2023-05-18

**Authors:** Ryan Guillemette, Matthew C. Harwell, Cheryl A. Brown

**Affiliations:** Pacific Coastal Ecology Branch, United States Environmental Protection Agency, Newport, Oregon, United States of America; The University of Akron, UNITED STATES

## Abstract

Rain contains encapsulated bacteria that can be transported over vast distances during relatively short periods of time. However, the ecological significance of bacteria in “precontact” rainwater–rainwater prior to contact with non-atmospheric surfaces–remains relatively undefined given the methodological challenges of studying low-abundance microbes in a natural assemblage. Here, we implement single-cell “click” chemistry in a novel application to detect the protein synthesis of bacteria in precontact rainwater samples as a measure of metabolic activity. Using epifluorescence microscopy, we find approximately 10^3^–10^4^ bacteria cells mL^-1^ with up to 7.2% of the observed cells actively synthesizing protein. Additionally, our measurement of less than 30 μM total organic carbon in the samples show that some rainwater bacteria can metabolize substrates in very low organic matter conditions, comparable to extremophiles in the deep ocean. Overall, our results raise new questions for the field of rainwater microbiology and may help inform efforts to develop quantitative microbial risk assessments for the appropriate use of harvested rainwater.

## Introduction

Rainwater microbiology is a relatively understudied field but has important implications for microbial biogeography [1,2], atmospheric science [1,3], and human health [4,5]. Previous studies show that “precontact” rainwater–rainwater prior to contact with non-atmospheric surfaces–contains bacterial abundances on the order of 10^2^–10^4^ cells mL^-1^, with few samples containing up to 10^5^ cells mL^-1^ [3,4,6]. The concentrations are similar to those found in cloud water [7] but approximately 1–3 orders of magnitude lower than concentrations found in most ocean [8] and freshwater environments [9]. Intriguingly, even at low abundance, rainwater samples contain phylogenetically diverse, viable, and active bacteria [4,10]. Furthermore, bacteria are dispersed by rain between coastal and inland sites [4,11], and harmful bacteria in atmospheric aerosols can potentially spread from certain regions to other habitats via rain [5,12]. It is important to understand the abundance and activity of dispersed bacteria because environmental bacteria help shape global ecosystems through their dominant role in biogeochemical cycling and ecological community structuring [8]. It is still unknown as to how, or to what extent, rainwater bacteria affect the habitats that they are deposited into through these various potential roles.

Rainwater microbiology also has significance for the health of humans and other organisms. Microbes, including some pathogens, have ubiquitous presence in the atmosphere and can be dispersed by natural methods like rain [5]. A potential human pathogen (*Acinetobacter johnsonii*) and *Escherichia coli*-like sequences were found in precontact rainwater samples collected in South Korea [4]. Additionally, the plant pathogen, *Pseudomonas syringae*, can serve as cloud condensation nuclei and undergo dispersal by rain [13–15]. While these studies demonstrate that the lifecycle of certain pathogens are influenced by rain, much remains to be learned about the epidemiological risks posed by rainwater bacteria. Furthermore, there have been increasing efforts to harvest potable and non-potable rainwater for human use and to better understand the quality and safety of the collected rain [16–18]. However, microbes and other substances (e.g., including dust, particles, nutrients, organic matter, etc.) in precontact rainwater are not currently considered in rainwater harvesting studies. As the base of harvested rainwater, precontact rainwater composition and microbial ecology are important parameters to consider when assessing the risks associated with rainwater storage and use.

However, despite being studied for decades, the field of rainwater microbiology has received remarkably little attention and made relatively limited progress. This may be due, in part, to historically limited methods available for analyzing aquatic samples that contain environmental microbes in low-abundance, such as rain. Here, we apply single-cell “click” chemistry to further evaluate precontact rainwater’s capacity to support metabolically active rainwater bacteria. We use a commercially available kit called Click-iT^TM^ L-Homopropargylglycine (HPG). Click chemistry methods, such as HPG, have been adapted for use in marine microbiology studies [19,20] but have not been broadly applied to other aquatic systems. Briefly, active cells within a sample incorporate a spiked methionine analog, HPG, into newly synthesized protein and the protein fluoresces green when observed with epifluorescence microscopy. The fluorescence is due to HPG’s alkyne-conjugation with a fluorophore (Alexa Fluor 488) resulting from the “click” reaction during sample processing.

In applying the click chemistry method, we concentrate cells from low-abundance samples and then quantify the proportion of metabolically active cells in rainwater for the first time. We also measure the concentration of organic matter in samples to gain insight into the substrate available for supporting the observed activity. The overall goals of this study are to: (1) assess the applicability of single-cell click chemistry for rainwater microbiology; and (2) to determine the extent of metabolically active bacteria in precontact rainwater. We discuss the implications of our findings within the context of general rainwater microbiology and the field of rainwater harvesting.

## Material & methods

### Sample site and rain collection protocol

Rainwater was collected on the roof of the U.S. Environmental Protection Agency’s laboratory in Newport, Oregon, USA in the early rainy season of 2021 on April 24, April 25, May 7, and May 26. Prior to each collection, three 20 L polycarbonate buckets were 3X acid washed (10% HCl) and Super-Q-water rinsed. They were then deployed during a rain event for 30 minutes to rinse (condition) the buckets, decanted, and left to collect rainwater for approximately five hours. Collected rainwater was pooled into one bucket and sub-samples for each analysis were taken with sterile serological pipettes in a laminar flow hood. In total, 0.5–3 liters of rainwater was collected per event. All samples were processed within two hours of field collection.

### Rainwater conditions

#### TOC analysis

Three 30 mL samples from each rain event were taken for TOC analysis. The rainwater was aliquoted into I-CHEM™ “EPA Pre-cleaned” borosilicate vials with cap and septa (Thermo Scientific, Waltham, MA, USA), and then frozen at -20° C. Samples were shipped on dry ice and analyzed at the Marine Chemistry Laboratory, University of Washington, Seattle. Analysis was conducted with a Shimadzu TOC-Vcsh carbon analyzer using the high temperature catalytic oxidation method (Standard Method 5310 B-00) and measured on a non-dispersive infrared detector. Samples were acidified (w/ 6N HCl) prior to organic carbon analysis then sparged and injected into the system. Samples were evaluated for statistical difference by using unpaired t tests.

For quality assurance, an internal reference standard (41.6 μM C) was included during analysis. The reference measured as 43 μM C, showing a Shimadzu instrument error of 1.4 μM C. Additionally, Super-Q-water controls were run to test whether the rainwater collection buckets leached TOC. During the April 24^th^ event, triplicate Super-Q-water samples were collected directly from the Super-Q dispenser and processed for TOC analysis as previously described. In parallel, the rainwater collection buckets were filled with Super-Q-water from which triplicate TOC samples were also taken. The 1.5 μM C difference in TOC between the dispensed Super-Q-water control (7.4 ± 0.4 μM C) and bucket-contacted Super-Q-water control (8.9 ± 0.0 μM C) was on the order of the Shimadzu instrument error and considered negligible.

#### Specific conductance and pH

Specific conductance was measured with a YSI 3200 Conductivity Instrument. An Orion Star A211 (Thermo Scientific) was used to measure pH. The pH probe was calibrated at 25° C using Oakton® pH buffer packs with pH of 4.01, 7.00, and 10.01 (Cole-Parmer, Vernon Hills, IL, USA).

#### Inorganic nutrient analysis

One 50 mL rainwater sample was taken from each rain event and frozen at -80° C. Samples were shipped on dry ice and analyzed at the Marine Chemistry Laboratory, University of Washington, Seattle. Standard methods for phosphate (365.5; rev. 1.4), nitrate and nitrite (353.4; rev. 2), ammonium (349.0), and silicic acid (366.0) measurement were conducted according to the UNESCO (1994) Protocols for the Joint Global Ocean Flux Study (JGOFS) Core Measurements, IOC Manual and Guides 29 [21].

#### Click chemistry & cell counts

Protein synthesizing single-cell bacteria were detected using click chemistry according to [19,20]. For each rain event, one 50 mL rainwater sample was incubated with 2 μM HPG (final concentration) for one hour at room temperature (RT) in the dark. Incubations were stopped by formalin fixation (2% final concentration) on ice and filtered through 0.2 μm pore-size GTTP polycarbonate membranes (Millipore Sigma, Burlington, MA, USA) to retain bacteria cells. Filters were air dried at RT then immediately processed with a Click-iT L-Homopropargylglycine reagent kit (Thermo Scientific) according to [19], with slight modification. Processing entailed placing the filter face-up onto a 50 μl drop of reaction cocktail and incubating in the dark at RT for 1 h. The freshly made cocktail consisted of a 77.5 μl aliquot of Super-Q-water first mixed with 10 ul of reaction buffer, 2 ul copper (II) sulfate, 0.5 μl Alexa Fluor 488, and finally 10 μl of buffer additive. Note that the Super-Q-water was filtered through a 0.02 μm pore-size Anodisc filter (Whatman, Buckinghamshire, UK) prior to mixing the cocktail to remove potential microbial contaminants. After incubation, the filter was washed twice with 0.02 μm filtered Super-Q-water, dabbed dry on absorbent paper, and air dried in the dark. The filter was then counter-stained and mounted with Vectashield® H-1200, a DAPI (4′,6-diamidino-2-phenylindole) containing antifade mounting medium (Vector Labs., Burlingame, CA, USA). In the aquatic sciences, bacteria cell abundance is commonly determined by direct cell counts using DAPI stain [22].

The sample filters were examined with a Zeiss Axioskop 20 epifluorescence microscope using a 100x objective. Peak channel excitation and emission (in nanometers) were 365 and 445/50 for DAPI-stained cells, and 475/40 and 530/50 for Alexa 488-labeled, HPG-positive cells. At least 100 DAPI-stained cells from 10 or more microscopic fields of view were counted for each sample. Cells that stained positive only for DAPI were scored as DAPI^+^ or “total cells”, while DAPI^+^ cells that were also HPG-labeled were scored as HPG^+^ or “active cells”. Counts were converted to cells mL^-1^ by using the appropriate magnification and dilution factors. Total cell abundance was reported as the mean ± standard deviation of DAPI^+^ counts per field of view, while active cells were reported as a percent of the total cell abundance (HPG^+^ cells / DAPI^+^ cells * 100). Samples were evaluated for statistical difference by using unpaired t tests.

The following controls were implemented during each sampling event. Two HPG controls were analyzed in parallel to ensure that: (1) the reagents worked (a positive control); and (2) that there was no non-specific HPG labeling of cells (negative-controls). The positive control was live estuary water from Yaquina Bay incubated with HPG. All the positive controls worked, as samples from each event contained at least 10^5^ HPG^+^ cells mL^-1^. The negative controls consisted of a rainwater sample and an estuary sample that were each formalin fixed prior to HPG incubation according to [19,20]. No HPG^+^ cells were detected in any of the negative controls. For procedural controls, a sample was taken from the final Super-Q-water rinse of each bucket to confirm that the buckets were not contaminated with bacteria prior to deployment. Cell abundances from the Super-Q-water rinse were always at least two orders of magnitude lower than our rainwater counts. Furthermore, since the buckets were pre-conditioned with rainwater for 30 minutes and decanted prior to sample collection any residual cells from the Super-Q-water rinse would be greatly diluted to a negligible quantity. We also examined a blank filter membrane from the package and a filter membrane that received a sample of 0.02 μm filtered Super-Q-water in the filtration setup. Both membranes were treated with the HPG reagents and DAPI (as above) to check for filter membrane, apparatus, or reagent contamination. No cells were observed in the blanks.

## Results

### Rainwater conditions

In general, rainwater conditions varied little between sampled rain events (Table 1). Phosphate, silicic acid, and nitrite measured 0.0 μM for all samples except phosphate on May 7^th^ (0.1 μM) and nitrite on May 26^th^ (0.9 μM). Nitrate ranged from 1.2–4.7 μM (mean, 3.1 ± 1.4 μM), while ammonium was between 1.5–6.0 μM (mean, 3.6 ± 2.3 μM). There was approximately 2-fold difference in TOC from May 26^th^ (29.3 ± 0.4) in comparison to TOC on April 24^th^ (17.3 ± 0.3; p < 0.001) and April 25^th^ (18.2 ± 0.0; p < 0.001). Total organic carbon was not sampled on May 7^th^ due to insufficient rainwater volume, and one sample each from April 25^th^ and May 26^th^ were discarded due to shipment damage. Specific conductance averaged 88.0 ± 61.6 μS/cm, with a range of 23.7–167.0 μS/cm. For all samples the pH was between 5.0–5.1.

**Table 1:** Total organic carbon, nutrients, pH, & specific conductance of Newport, OR rainwater samples.

Date	TOC	PO_4_	Si(OH)_4_	NO_3_	NO_2_	NH_4_	pH	Conductance
	(μM)	(μM)						(μS/cm)
April 24, 2021	17.3 ± 0.3	0.0	0.0	1.2	0.0	1.2	5.0	167.0
April 25, 2021	18.2 ± 0.0	0.0	0.0	3.1	0.0	2.2	5.1	23.7
May 7, 2021	NS	0.1	0.0	4.7	0.0	6.0	5.0	59.5
May 26, 2021	29.3 ± 0.4	0.0	0.0	3.3	0.9	5.0	5.1	102.0

TOC values reported as mean ± SD of two replicates, except 4/24/2021 (3 replicates).

All other values are from measurement of one sample.

(NS) = Not Sampled.

### Bacteria abundance and activity

Total bacteria abundance (DAPI^+^ cells) in rainwater samples ranged from 5.3 ± 1.4 x 10^3^–1.5 ± 3.6 x 10^4^ cells mL^-1^ (mean, 8.3 ± 4.6 x 10^3^ cells mL^-1^; median, 6.5 x 10^3^ cells mL^-1^) (Table 2). The percent of active cells (HPG^+^) ranged from approximately 1–2% in samples from April 24^th^, April 25^th^, and May 7^th^ (Table 2). In rainwater from May 26^th^, we observed 7.4% of the population as active and the total bacteria abundance was 2–3-fold higher (p < 0.01) in comparison to all other samples in this study.

**Table 2:** Total bacteria abundance (DAPI^+^ cells) and the percent of active cells (HPG^+^) in Newport, OR rainwater samples.

Date	Total bacteria	Active cells
	(10^3^ cells mL^-1^)	(%)
April 24, 2021	5.5 ± 1.4	1.1
April 25, 2021	5.3 ± 1.4	2.3
May 7, 2021	7.4 ± 1.8	1.7
May 26, 2021	15 ± 3.6	7.2

## Discussion

### Utility of single-cell techniques in rainwater microbiology

Our single-cell click chemistry application represents the first quantification of metabolically active bacteria cells in precontact rainwater. This contrasts with previous rainwater studies which have either measured bacterial activity in bulk [4] or quantified the number of viable cells [10]. For example, Cho & Jang (2011) provided the first direct metabolic activity measurements of bacteria in precontact rainwater by using ATP measurement [4]. They found 3.0 ng and 0.5 ng ATP L^-1^ in two samples and extrapolated the values with cell abundance data to estimate 1.9 and 5.0 fg ATP cell^-1^, respectively. The strength of their ATP measurement method was its high sensitivity and that the values allow for estimation of bacterial biomass, an important metric in aquatic microbial ecology and biogeochemical cycling [23] (also see [24] and references therein). However, while averaging bulk activity (e.g., ATP) among the total cells in a sample is convenient for conceptualization purposes, it overestimates per-cell activity for most cells and underestimates it for the proportion that are in fact active. In our samples, we found that 1.1%– 7.2% of the cell populations were active, so averaging bulk activity over the whole population would have underestimated per cell activity by up to 99%. Future rainwater studies might consider using multiple methods in tandem (e.g., bulk, and single-cell analyses) so that the abundance and proportion of active cells can be quantified along with the quantity of biomass produced.

Additionally, our quantification of active cells represents: (1) an estimate for the number of metabolizing cells in falling rain that were presumably utilizing organic matter in the atmosphere; and (2) the number and proportion of cells that were active when deposited by rain into a new habitat. It is noteworthy that the latter implication might be advantageous for the proliferation and survival of individual cells and specific taxa upon deposition. Indeed, Cho & Jang (2011) found that rare taxa in precontact rainwater samples (taxa comprising <1% of detected pyrosequencing reads) comprised between 22.2% - 60.2% of the community after 13–18 hr laboratory incubation [4]. It is possible that many cells in their study might have been initially inactive at T_zero_ but those that were active grew rapidly–like our study where most cells (93% - 99%) were inactive. This suggests that the active cells, even at relatively low abundance might have been primed to grow quickly over time.

This study also reinforces the known utility of click chemistry for in situ analysis of low microbial abundance samples [25]. The ability to filter cells onto a membrane allows researchers to concentrate the cells in a sample that are then suitable for analysis with microscopy, effectively bypassing other activity methods that require large volumes such as ATP (requires approximately 2 L of rainwater [4]) or methods that use radioisotopes, like conventional techniques for protein and DNA synthesis measurement [26,27]. Furthermore, click chemistry is a highly adaptable method that can be used in combination with other single-cell techniques. For example, researchers have used molecular probes that bind to specific nucleic acid sequences (fluorescence in situ hybridization; FISH) with HPG to identify active taxa in aquatic samples [20], which might be highly effective for rainwater studies since bacterial community analysis requires large volumes of rainwater. Future studies could also use click chemistry (e.g., HPG) coupled with FISH to determine the active bacterial taxa immediately upon rainwater collection and then take more samples over time to determine which taxa survive and proliferate.

### Bacterial growth dynamics in low TOC rainwater

Another intriguing finding was our observation that bacteria were active in samples with relatively low TOC. Total organic carbon is comprised of a dissolved (DOC) and particulate (POC) phase and is often used as a proxy measurement for organic matter concentration in aquatic studies (as reviewed in [28]). Furthermore, the quality and quantity of TOC is known to influence the growth [29] and community composition [30,31] of aquatic bacteria. Measurements of precontact rainwater TOC (or DOC) are sparse, with reported values from ten studies ranging from 10.0 μM C– 779.0 μM C (see S1 Table and Siudek at al. 2015 [32]). Our measurements (17.3–29.3 μM TOC) are within the lower range of reported values, and well below the typical values reported for other aquatic systems. As reference, a common standard for marine surface water organic carbon is 75 μM, and 45 μM for the deep sea [33]. Additionally, a compilation of global TOC values from freshwater lake studies (n = 8,300 lakes) showed a mean of 464.4 ± 233.1 μM C, with a lower 95% confidence interval of 236.2 μM C [34]. Reports on cloud water TOC also show comparatively higher TOC concentrations. For example, a 10-year study at Puy de Dome, France measured TOC values ranging from 83 μM C to over 2,000 μM C (median, 250 μM C) for 199 samples taken from 73 cloud events [35].

To our knowledge, similar observations of bacterial activity in low TOC water samples have only been reported for bacteria in the deep sea. Peoples et al. (2018) found using HPG that ~18% of bacteria cells were active in the abyssal and hadal zones of the Mariana Trench [25] where typical TOC values are ~40 μM C [36]. This is like the relatively low activity of bacteria we found in our samples, with comparable though slightly higher TOC concentrations, and similar total bacteria abundances (10^4^ cells mL^-1^). By contrast, multiple activity measurements with HPG in the surface ocean show much higher proportions of active cells (75%, [25]; 66.1%, [19]) in waters that contain approximately 70 μM TOC and bacterial densities on the order of 10^6^ cells mL^-1^ [37]. Interestingly, in somewhat of a paradox, cloud water bacteria from relatively high TOC water at Puy de Dome (referenced above) had high proportions of cell activity (possibly 100%) based on estimates from ATP measurement, yet the cell abundances never exceeded 10^5^ cells mL^-1^. However, it was believed that the relatively low cloud bacteria abundances were due to growth (cell division) inhibitions from toxic air pollutants in the measured TOC [7].

While not tested here, it is possible that TOC quality and concentration might have regulated bacterial abundance and activity in our samples. The subject has not been explored in rainwater microbiology but extensive data supporting this argument exists for other aquatic systems. It is well established that most aquatic organic matter (> 90%) exists in a refractory state that is biologically unavailable to bacteria [38] and that bacteria generally use a small fraction of the available organic matter to produce biomass, with most of the organic carbon being used for cellular respiration versus cellular production (see bacterial growth efficiency [39]). In fact, studies of highly oligotrophic waters (like our rainwater samples) show up to 99% of the consumed carbon may be utilized for respiration [39,40]. If we use these data as assumptions in our study and consider that no new organic matter was produced in our collected samples (no cells with auto-fluorescing photosynthetic pigments were observed), then a very small fraction of TOC remains for bacterial growth. Within this context, it is noteworthy that TOC from May 26 was 2-fold higher than in our other measured samples, as was bacterial abundance (2–3-fold) and activity (3–7-fold). This may suggest that certain rainwater bacteria taxa are better adapted for growth in low TOC water.

### Potential sources of bacteria in collected rainwater

Throughout our study care was taken to ensure that rainwater did not contact other surfaces and become contaminated prior to deposition into the open collection buckets. However, the full range of variability that drives the microbial content of collected rainwater is complex and includes confounding variables such as atmospheric deposition of aerosols (including bacteria), wind speed and direction governing aerosol sources, fog, and perhaps other factors [41]. Therefore, it’s possible that our analyses included bacteria from sources other than raindrops. For example, a study off the coast of Maine found significant bacteria in collected coastal fog, which taxonomically aligned with microbial communities in the near surface ocean and that microbial fallout greatly increased with fog [42,43]. Within this context, one plausible explanation for the observed increase in bacteria abundance and activity on May 26 could have been from fog deposition. Local weather observations noted fog throughout our sampling on May 26, while no fog was reported for April 24, April 25, or May 7 [44,45]. Additionally, studies have shown that sea spray can aerosolize microbes and aerosolized microbes can travel before terrestrial deposition [46–48]. It would be interesting to know if any sea spray aerosols, including bacteria and perhaps organic matter were deposited into the sampling vessels, as typical coastal ocean values near the collection site are on the order 10^6^ bacteria cells mL^-1^ and over 100 μM TOC [49,50]. Our small sample size also means we likely missed the full range of parameter values for the region. Sampling at more geographical sites and at a greater frequency could better inform the range of values, as could measuring bacteria abundance in the aerosols before and after rain events. Studies show that aerosolized bacteria are scavenged by rain [5] so knowing the before and after concentrations could help inform the source of rain bacteria. While addressing each of these discussed scenarios was beyond the scope of our study, they could have contributed to the rainwater parameter values that we measured and may have influenced the range of values over space and time for the region.

### Future directions and conclusion

The timing seems ripe for advancing rainwater microbiology given the recent availability of more single-cell and molecular techniques. Additionally, researchers might benefit from continued method improvements for low rainwater volume and low bacterial abundance analyses, as well as sampling protocols that allow for better discernment between bacteria sources in collected rainwater (e.g. raindrops vs aerosols vs fog, etc.). Another study that warrants consideration would be to test if the impact of rain deposition, or any sheer forces associated with the impact, may affect the survivability of rain deposited bacteria. More in-depth studies on the growth requirements for rainwater bacteria are also needed. Researchers could use flow cytometry or suspended microchannel resonator devices to conduct high-throughput bacterial physiology experiments with rainwater isolates under various conditions. This could include a range of natural organic matter concentrations, as well as model organic substrates to define the minimal growth requirements of select bacterial isolates and the carrying capacity of field collected rainwater itself. Such experiments, in combination with single-cell microscopic analyses should provide increased fundamental knowledge for general and applied rainwater microbiologists.

Furthermore, water resource scientists and decision makers could also benefit from further development of this field. As discussed in Alja’fari et al. (2022), rainwater harvesting (or roof runoff) may be an underutilized alternative water source in the US [51] and there is insufficient data on the microbial water quality of collected rain, making quantitative microbial risk assessments (QMRA) difficult [16]. Such notions regarding QMRA have been raised previously [17] and a review by Ahmed et al. 2011 concluded that based on published data “the microbial quality of [roof harvested rainwater] should be considered potentially poor until more rigorous microbial assessment can be undertaken” [52]. In general, we agree with these assessments. However, we also recommend the inclusion of microbial water quality data for precontact rainwater in the efforts to develop both QMRAs and guidelines for using harvested rainwater. Such data would provide a baseline of what eventually contacts the roof and becomes contaminated, or altered, during collection. The significance of this would be to determine not only what microbes are coming from the atmosphere versus other sources (fecal matter, roof-debris, etc.) but also the source and concentration of nutrient and organic matter available for bacterial growth. Since the rainwater collection source (geographical location and proximity to industry) might play a role in the organic matter concentration of rain (S1 Table; [32]) it might be useful for engineers and architects to consider building rainwater collection systems that bypass contaminated roofs altogether (e.g., an artificially constructed catchment [53]). In this way, relatively pristine rainwater with low bacteria and low TOC might need less treatment and could potentially be used for more purposes. A future synthesis of pre- and post-contact rainwater studies from the same locations could help determine the significance of precontact rainwater quality for developing risk and health assessments.

In conclusion, our study demonstrates that single-cell click chemistry is a useful tool in quantifying active bacteria in rainwater and that some rainwater bacteria are well adapted to low organic matter conditions. It also suggests that organic matter concentration might regulate the overall carrying capacity of bacteria in rainwater, as has been shown in other aquatic systems. Future studies may benefit from the implementation of these techniques to further understand the ecophysiology of rainwater bacteria, which may affect the general ecology of natural systems and influence human health.

## Supporting information

S1 Table Modified table from Siudek et al. 2015 [32]. Summary of studies reporting organic carbon analysis of precontact rainwater; sorted in ascending order (Concentration; μM).
(DOCX)
