# Supplementary material for: Metabolically active bacteria detected with click chemistry in low organic matter rainwater
Source: PLoS One. 2023 May 18;18(5):e0285816. doi: 10.1371/journal.pone.0285816 (PMC10194877; doi:10.1371/journal.pone.0285816)
Supplement: S1 Table — Summary of studies reporting organic carbon analysis of precontact rainwater; sorted in ascending order (Concentration; μM). (DOCX) [file pone.0285816.s001.docx]

**Supporting information**

**S1 Table.** **Modified table from Siudek et al. 2015.** Summary of studies reporting organic carbon analysis of precontact rainwater; sorted in ascending order (Concentration; µM).

| **Source** | | **Organic Carbon** | **Location** | **Setting** | |
| --- | --- | --- | --- | --- | --- |
|  |  | **(µM)** |  |  | |
| Current Study |  | 17.3 – 29.3* | Newport, OR, USA | | Coastal |
| Kieber et al. | 2002 | 10.0 – 400.0^ | Dunedin, New Zealand | | Coastal |
| Yan & Kim | 2012 | 15.0 – 779.0^ | Seoul, Korea | | Urban |
| Kieber et al. | 2002 | 58.3^ | Dunedin, New Zealand | | Coastal |
| McDowell & Likens | 1988 | 91.6^ | New Hampshire, USA | | Forest |
| Pan et al. | 2010 | 200.0 – 325.0^ | Northern China | | Various |
| Quideau & Bockheim | 1997 | 241.4^ | Wisconsin, USA | | Forest |
| Coelho et al. | 2008 | 338.0^ | Araraquara, Brasil | | Suburban |
| Liu & Sheu | 2003 | 391.3^ | Guandaushi, Taiwan | | Subtropical forest |
| Siudek et al. | 2015 | 393.0^ | Jeziory, Poland | | Regional |
| Siudek et al. | 2015 | 424.6^ | Poznań, Poland | | Urban |

(*) Total organic carbon

(^) Dissolved organic carbon
